# Transmission of SARS-CoV-2 Delta variant from an infected aircrew member on a short-haul domestic flight, Australia 2021

**DOI:** 10.1093/jtm/taac144

**Published:** 2022-11-30

**Authors:** Kirsten M Williamson, Michelle Butler, Benjamin Elton, Joanne Taylor, Fakhrul Islam, Michael P Douglas, Martyn D Kirk, David N Durrheim

**Affiliations:** Hunter New England Population Health, Hunter New England Local Health District, Newcastle, NSW 2305, Australia; Hunter New England Population Health, Hunter New England Local Health District, Newcastle, NSW 2305, Australia; Hunter New England Population Health, Hunter New England Local Health District, Newcastle, NSW 2305, Australia; Hunter New England Population Health, Hunter New England Local Health District, Newcastle, NSW 2305, Australia; School of Medicine and Public Health, University of Newcastle, Newcastle, NSW 2308, Australia; Hunter New England Population Health, Hunter New England Local Health District, Newcastle, NSW 2305, Australia; Public Health Response Branch, New South Wales Ministry of Health, Sydney, NSW 2060, Australia; School of Medicine, University of Western Sydney, NSW 2052, Australia; National Centre for Epidemiology and Population Health, Australian National University, Canberra, ACT 2601, Australia; Hunter New England Population Health, Hunter New England Local Health District, Newcastle, NSW 2305, Australia; School of Medicine and Public Health, University of Newcastle, Newcastle, NSW 2308, Australia

**Keywords:** COVID-19, SARS-CoV-2, Delta, variant, flight, aircraft, transmission, mask

## Abstract

In June 2021, when COVID-19 incidence in Australia was low, a COVID-19 (Delta variant) cluster occurred on an 81-minute domestic flight, with an aircrew member as the likely source. Outbreak investigation demonstrated that SARS-CoV-2 may be transmitted during short-haul flights and that mask use protected against infection.

On 26 June 2021, Australian public health authorities were notified of an aircrew member who tested positive for Severe Acute Respiratory Syndrome Coronavirus-2 (SARS-CoV-2) Delta variant of concern. They had worked earlier that day on a short, domestic flight from the Gold Coast, Queensland to Sydney, NSW. At this time, COVID-19 incidence in Australia was low, with a total of 257 locally acquired cases (1.00 cases per 100 000 population) notified nationally between 1 January and 20 June 2021.^1^ National control measures included: international border closures; interstate border restrictions; compulsory face masks within airports and aircraft; mandatory notification, isolation and contact tracing of all cases; and 14-day quarantine and serial SARS-CoV-2 polymerase chain reaction (PCR) testing of all close contacts.^1–3^

Urgent contact tracing occurred within 24 hours of the flight, with all aircrew and passengers deemed close contacts. Subsequently several COVID-19 cases were identified amongst passengers. An outbreak investigation and cohort study were conducted under the NSW *Public Health Act 2010* to identify potential risk factors for SARS-CoV-2 transmission during and around the flight.

Outbreak cases were defined as persons confirmed with COVID-19 by validated PCR^2^; AND travelled on the Gold Coast to Sydney flight on 26 June 2021 OR were close contacts of cases from the flight; AND had an infection onset between 12 June and 24 July 2021. Cases were interviewed using a structured hypothesis-generating questionnaire (Supplementary Materials, S1). Non-cases were invited to complete a short, online Research Electronic Data Capture survey^4^ (Supplementary Materials, S2). Analyses were conducted in SAS Enterprise Guide v7.1 and STATA 14.2.

The 81-min flight on-board a Boeing 737-800 aircraft carried 6 aircrew and 139 passengers. Seven of eight business class seats were occupied (87.5%) and 130 of 168 economy seats were occupied by 132 passengers (78.6%) (Figure 1A).

**Figure 1:**
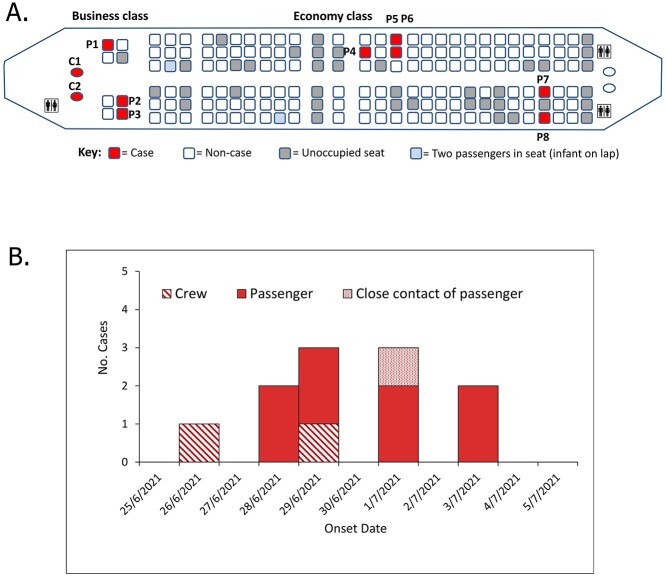
(A) Cabin map and COVID-19 case locations and (B) epidemic curve of COVID-19 cases associated with Gold Coast to Sydney flight on 26 June 2021.

In total, 11 COVID-19 cases were associated with the flight (2 aircrew, 8 passengers, 1 close contact of a passenger case), giving a primary attack rate of 6.9% (10/145). The median case age was 41 years (range: 16–79), seven (64%) were female and none (0%) had received a COVID-19 vaccine dose. Onsets of infection ranged from 26 June to 3 July 2021, peaking 3–5 days after the flight (Figure 1B). Of 11 case specimens, 10 underwent whole genome sequencing (WGS) and found an identical SARS-CoV-2 Delta variant sub-lineage (B.1.617.2) within two single nucleotide polymorphism differences from each other.

The index case (C1), an aircrew member, primarily worked at the front of the cabin, serving food and drink to the pilots and business class passengers. Prior to boarding, C1 cleaned the front cabin area and toilets, and prepared drinks in the front galley while passengers boarded; thus, C1 did not have contact with passengers outside of the aircraft. C1 wore an airline-issued fabric mask with filter but removed it to make PA announcements, in accordance with airline safety regulations at the time. After the flight, C1 did not go to the baggage carousel but exited the terminal directly. C1 reported developing symptoms towards the end of the flight, and tested positive for SARS-CoV-2 on urgent PCR that evening. The second crew case (C2) also worked in the front half of the cabin. C2 wore a disposable surgical mask, however, in keeping with the airline policy, C1 and C2 removed their masks while preparing the cabin before the flight. C2 initially tested negative on SARS-CoV-2 PCR in the evening of 26 June, but tested positive and developed symptoms on 28 and 29 June 2021, respectively.

Of the eight passenger cases, one travelled alone, four travelled as pairs and three as a group. Three sat in business class, three in the middle of economy and two at the rear. All reported removing their masks during the flight. All tested negative on SARS-CoV-2 PCR on 27 June, but subsequently developed symptoms and tested positive between 28 June and 3 July 2021. Given some cases were household members, it could not be determined if three had acquired their infection during the flight or from their co-traveller afterwards (Supplementary Materials, S3). Upstream tracing found the index case (C1) was the only case with a known COVID-19 exposure prior to the flight. All other (non-case) crew and passengers tested negative on PCR within 24 hours of disembarkation and remained negative on serial PCR testing (days 7 and 12) during their 14-day quarantine.

Of 139 passengers, 113 (81.3%) passengers (8 cases and 105 non-cases) completed surveys. Of these 113, 3 (2.7%) wore N95/P2 masks, 60 (53.1%) wore surgical masks, 4 (3.5%) wore cloth masks with filter, 31 (27.4%) wore cloth masks and 15 (13.3%) did not specify type. In sum, 73 (64.6%) passengers reported removing their masks during the flight, mostly to eat or drink (71.2% [52/73]).

Factors associated with increased risk of passengers becoming a case included: removal of mask during flight (*P* = 0.03), including to eat (RR 5.3, *P* < 0.01) and drink (RR 10.6, *P* < 0.01); and sitting in business class (RR 9.1, *P* < 0.001) where the index case predominately worked. Relative risks could not be calculated for mask removal due to zeros in cells (Table 1).

**Table 1:** Potential risk factors for COVID-19 in passengers on the Gold Coast to Sydney flight on 26 June 2021

		**Exposed**			**Unexposed**				
**Variable**	**Responses**	**Total**	**No. cases**	**Attack rate (%)**	**Total**	**No. cases**	**Attack rate (%)**	**Relative risk**	***P* value**
**Total Passengers**	**139**								
**Vaccination Status**	Received 1+ dose of COVID-19 vaccine *(1 dose, 2 doses)*	26 *(17,9)*	0	0.0	113	8	7.1		0.16
**Completed Survey**	113								
**Mask wearing**	**Removed mask at any time during flight**	73	8	11.0	40	0	0.0		**0.03**
	**Removed mask to eat**	27	5	18.5	86	3	3.5	**5.3**	**<0.01**
	**Removed mask to drink**	45	7	15.6	68	1	1.5	**10.6**	**<0.01**
	**Removed mask to eat or drink**	52	8	15.4	61	0	0.0		**<0.01**
**Seating**	Window seat	42	4	9.5	70	4	5.7	1.7	0.45
	Aisle seat	36	2	5.6	76	6	7.9	0.7	0.65
	Middle seat	34	2	5.9	78	6	7.7	0.8	0.73
	**Business Class seat**	7	3	42.9	106	5	4.7	**9.1**	**<0.001**
	Rear of plane (Rows 14–30)	64	5	7.8	49	3	6.1	1.3	0.73
**Entry Door**	Entered via front door	65	5	7.7	45	0	0.0		0.06
**Exit Door**	Exited via front door	64	3	4.7	46	2	4.3	1.1	0.93
**Bathroom**	Used bathroom during flight	10	1	10.0	100	4	4.0	2.5	0.39
**Hand Sanitizer**	Did not use during flight	62	3	4.8	46	0	0.0		0.13

Note: Chi-squared test used to calculate *P* values; *P* ≤ 0.05 considered statistically significant (bold). Relative risks could not be calculated where cells equal zero.

Sensitivity analysis (three cases removed where acquisition from the co-traveller could not be excluded) found risk increased when masks were removed to eat (RR 12.9, *P* < 0.01), and if seated in a business class (RR 22.1, *P* < 0.0001). However, mask removal without eating or drinking was no longer significant (*P* = 0.08) (Supplementary Materials, S4).

SARS-CoV-2 transmission has previously been reported during air travel, however. mostly on long-haul international flights between passengers.^5^^,^^6^ This COVID-19 cluster investigation on a short-haul, domestic flight found an aircrew member as the primary case and likely outbreak source. The initial negative results in all other cases, identical sub-lineages on WGS and low community incidence support this theory, as does the increased risk in business class where the index case primarily worked. The index case assisted briefly at the rear of the aircraft and all passenger cases entered or exited via the front doors, which could account for transmission to economy class passengers. There was no known contact between the index case and other cases (apart from C2) before or after the flight. Thus, despite measures employed to reduce respiratory pathogen transmission risk (including engineered air flow and HEPA filters),^7^ this investigation strongly supports SARS-CoV-2 transmission occurred on-board the aircraft.

Consistent mask use reduced passengers’ risk of COVID-19, which is consistent with other studies.^5^^,^^6^^,^^8^^,^^9^ Risk reduction appears greater if the infectious person is wearing a mask, and further reduced if exposed persons also wear masks.^9^ Thus, mask removal by the index case during PA announcements may have promoted transmission.

Some limitations should be considered. Symptom onset, source ascertainment and behaviours/activities relied upon self-reported information. Only univariate analysis could be conducted due to small case numbers. Strengths include high case ascertainment due to serial PCR testing, and high survey response rate.

This was the first COVID-19 outbreak associated with an Australian domestic flight, where an aircrew member was the likely source. The investigation demonstrated that SARS-CoV-2 may be transmitted during short-haul flights and that consistent mask use was protective against infection. Wearing masks on-board flights remains a sensible measure, especially in light of subsequent, highly transmissible variants where available vaccines may be less effective.^10^

## Supplementary Material

List_of_Supplementary_Materials_SARS-CoV-2_transmission_taac144

Supplementary_Material_S1_SARS-CoV-2_HGQ_taac144

Supplementary_Material_S2_SARS-CoV-2_taac144

Supplementary_Material_S3_SARS-CoV-2_taac144

Supplementary_Material_S4_SARS-CoV-2_taac144

## Data Availability

De-identified, raw data can be made available by the authors on request.
