# Supplementary material for: Transmission of SARS-CoV-2 Delta variant from an infected aircrew member on a short-haul domestic flight, Australia 2021
Source: J Travel Med. 2022 Nov 30;29(8):taac144. doi: 10.1093/jtm/taac144 (PMC9793396; doi:10.1093/jtm/taac144)
Supplement: List_of_Supplementary_Materials_SARS-CoV-2_transmission_taac144 [file list_of_supplementary_materials_sars-cov-2_transmission_taac144.docx]

**SUPPLEMENTARY MATERIAL**

**Supplementary Material S1:** Hypothesis generating questionnaire for passenger cases associated with Gold Coast to Sydney flight on 26 June 2021

**Supplementary Material S2:** REDCap online survey for passengers of Gold Coast to Sydney flight on 26 June 2021

**Figure S3: A)** Scenario One, where an infected crew member transmitted SARS-CoV-2 to five passenger cases and one air crew member case, with subsequent spread from infected passengers to four close contacts. **B)** Scenario Two, where an infected air crew member transmitted SARS-CoV-2 to eight passengers and one crew member, with subsequent spread to one close contact.

**Table S4:** Sensitivity analyses of potential risk factors for case and non-case passengers of Gold Coast to Sydney flight on 26 June 2021.
