# Supplementary material for: Transmission of SARS-CoV-2 Delta variant from an infected aircrew member on a short-haul domestic flight, Australia 2021
Source: J Travel Med. 2022 Nov 30;29(8):taac144. doi: 10.1093/jtm/taac144 (PMC9793396; doi:10.1093/jtm/taac144)
Supplement: Supplementary_Material_S1_SARS-CoV-2_HGQ_taac144 [file supplementary_material_s1_sars-cov-2_hgq_taac144.pdf]

**Passenger - Demographics**				
Name:				
Gender:	Female	Male	Non-binary	Declined
Symptom onset date:				
Vaccinated:	Yes	No	Don’t know	
Comments:<br>(including type and dose)				
Flight name:				
Seat number on boarding pass:				
Did you sit in this seat:	Yes	No	Don’t know	
Comments:				
Did you travel with others:	Yes	No	Don’t know	
Comments:<br>(Names of travelling party, seat number(s) if known)				
**Pre-flight**				
How did you check-in for your flight:	Online	Airline desk	Don't know	
Comments:				
Did you have checked luggage:	Yes	No	Don't know	
Comments:				
Did you use a digital boarding pass:	Yes	No	Don't know	
Comments:				
Did you visit the airport lounge pre-flight:	Yes	No	Don't know	
Comments:<br>(time spent in lounge in minutes)				
Did you use the bathrooms or make any purchases at a cafés etc. within the airport pre-flight:	Yes	No	Don't know	
Comments:				
Approximate time spent at gate pre-flight (in minutes)<br>Comments:				

**Boarding & seating**				
Did you wear a mask during the flight:	Yes	No	Don't know	
What type of mask did you wear:	Surgical	Cloth	Other	Don't know
Comments:				
Did you wear the mask for the whole flight:	Yes	No	Don't know	
Comments:				
Did you have your boarding pass scanned by a ground crew member before leaving the terminal:	Yes	No	Don't know	
Comments:				
To access the plane did you use a passenger tunnel or walk across the tarmac?	Tunnel	Tarmac	Don't know	
Comments:				
Which door of the plane did you enter via:	Front	Rear	Don't know	
Comments:				
Did you have to stand in line while waiting to board the plane, after having your boarding pass scanned:	Yes	No	Don't know	
Comments:				

Was social/physical distancing being practiced:	Yes	No	Don't know
Comments:			
Were you greeted by a cabin crew member upon boarding the plane:	Yes	No	Don't know
Comments:<br>(including crew name/description if known)			
Did you have carry-on luggage:	Yes	No	Don't know
Did you place your carry-on luggage in the over-head locker near your seat or elsewhere in the plane:	Near seat	Elsewhere	Don't know
Comments:			
Did a flight attendant assist you with placing your carry-on in the over-head locker:	Yes	No	Don't know
Comments:			
Did you travel with a child	Yes	No	Don't know
Did this child have their own seat or were they travel on your lap	Own seat	lap	Don't know
Did a flight attendant assist you with lap restraint seat beat for the child:	Yes	No	Don't know
Comments:			
Were you seated in an emergency exit row:	Yes	No	Don't know

Did a flight attendant conduct pre-flight consent check/exit row information with you:	Yes	No	Don't know
Comments:			
Did wait on-board of an extended time prior to take off (E.G. waiting for a late arriving passenger etc.):	Yes	No	Don't know
Comments:			
Were you seated near a flight attendant when they went through the safety demonstration (seat belts, life jackets etc.):	Yes	No	Don't know
Comments:			
Did a flight attendant interact with you while conducting the pre-take off safety check (bags under seat, tray tables, windows etc.):	Yes	No	Don't know
Comments:<br>(including crew name/description if known)			
**During flight**			
Did you remove your mask at any point during the flight:	Yes	No	Don't know
Comments:			
Did you buzz/call for assistance during the flight:	Yes	No	Don't know
Comments:			

Did you purchase any food, drinks or other during the flight:	Yes	No	Don't know
Comments:<br>(including payment method)			
Did you have the over-head fan on during the flight:	Yes	No	Don’t know
Comments:			
Did you use the toilet during the flight:	Yes	No	Don't know
Comments:<br>(including toilet location front/rear)			
Did you need to stand in line to wait for the toilet:	Yes	No	Don't know
Comments:<br>(including if others were standing and waiting at the same time)			
Did you read/handle any in-flight materials (E.G. safety cards, menus, magazines):	Yes	No	Don't know
Comments:			
Did a flight attendant interact with you while conducting the pre-landing safety check (declarations, bags under seat, tray tables, windows etc.):	Yes	No	Don't know
Comments:			

Did any other passengers outside of your travelling party interact with you during the flight:	Yes	No	Don't know
Comments:			
Did you leave your seat at any other point during the flight:	Yes	No	Don't know
Comments:			
**Exiting the plane**			
Do you recall having to wait for an extended prior of time after landing before being allow to exit the plane:	Yes	No	Don't know
Comments:			
Did you stand up as soon as it was safe to remove seatbelts:	Yes	No	Don't know
Comments:			
Did others reach over you to collect carry-on luggage from the over-head locker:	Yes	No	Don't know
Comments:			
Did you have to reach over others to collect carry-on luggage from the over-head locker:	Yes	No	Don't know
Comments:			
Who was standing near you when you were waiting to exit:	Other passengers	Members of travelling party	Don't know
Comments:			

Was social/physical distancing being practiced:	Yes	No	Don't know
Comments:			
Which door of the plane did you exit via:	Front	Rear	Don't know
Comments:			
To exit the plane did you use a passenger tunnel or walk across the tarmac?	Tunnel	Tarmac	Don't know
Comments:			
Were you farewelled by a cabin crew member upon exiting the plane:	Yes	No	Don't know
Comments:			
**Post-flight**			
Did you transfer directly onto another flight:	Yes	No	Don't know
Comments:			
Did you collect checked luggage from the carrousel:	Yes	No	Don't know
Comments:			
Was social/physical distancing being practiced:	Yes	No	Don't know
Comments:			

Did you observe anyone, including passengers and staff, before, during or after the flight with symptoms (did they look sick):	Yes	No	Don't know	
Comments:				
At what point did you remove your mask:	Comments:			
Did you assist anyone else remove their mask:	Yes	No	Don't know	
Comments:				
Did you use the bathrooms or make any purchases at a cafés etc. within the airport post-flight:	Yes	No	Don't know	
Comments:				
How did you travel from the airport to your home/place of residence:	Private car	Ride sharing / taxi	Public transport	Unknown / other
Comments:				
Did you visit any other venues on transit to your home/place of residence:	Yes	No	Don't know	
Comments:				
