# Supplementary material for: Transmission of SARS-CoV-2 Delta variant from an infected aircrew member on a short-haul domestic flight, Australia 2021
Source: J Travel Med. 2022 Nov 30;29(8):taac144. doi: 10.1093/jtm/taac144 (PMC9793396; doi:10.1093/jtm/taac144)
Supplement: Supplementary_Material_S2_SARS-CoV-2_taac144 [file supplementary_material_s2_sars-cov-2_taac144.pdf]

Passenger Survey

Dear [first_name] of seat [seat_no]

Thankyou for taking the time to complete the survey below. The information you provide will help us better understand COVID-19 transmission events and may help us identifiy ways to prevent cases of illness on flights in future.

Is this questionnaire being completed for a confirmed case?	◯ Yes<br>◯ No
Did you sit in your allocated seat?	◯ Yes<br>◯ No
Which seat did you sit in?	__________________________
Did you wear a mask on the plane?	◯ Yes<br>◯ No
What type of mask did you wear?	◯ Basic cloth mask<br>◯ Cloth mask with filter<br>◯ Surgical mask (i.e. blue disposable mask)<br>◯ Respirator (i.e. N95/P2)<br>◯ Other
Please describe the type of mask you wore	__________________________
Did you remove or lift up your mask while on the plane for any of the following reasons: (select all that apply)	☐ To Eat<br>☐ To Drink<br>☐ To Speak to someone<br>☐ While using the bathroom<br>☐ For comfort<br>☐ Other
Other (please specify)	__________________________
Please estimate the total time (in minutes) you were NOT wearing a mask during the flight (total flight duration was 90 minutes):	__________________________
Did you use hand sanitizer on the flight?	◯ Yes<br>◯ No
When on the plane did any flight crew speak to you whilst NOT wearing a mask fully covering their nose and mouth?	◯ Yes<br>◯ No
Please select all scenarios when flight crew who were NOT wearing a mask spoke to you	☐ When boarding the flight<br>☐ When exiting the flight<br>☐ To order or receive food/drink<br>☐ Assistance with overhead baggage<br>☐ Assistance with seatbelt or restraint<br>☐ Other

Other (please specify)	______________________________
When on the plane did any flight crew speak to you whilst YOU were NOT wearing a mask fully covering your nose and mouth?	◯ Yes<br>◯ No
Please select all scenarios when flight crew spoke to you whilst YOU were NOT wearing a mask	☐ When boarding the flight<br>☐ When exiting the flight<br>☐ To order or receive food/drink<br>☐ Assistance with overhead baggage<br>☐ Assistance with seatbelt or restraint<br>☐ Other
Other (please specify)	______________________________
Did you use the bathroom whilst on the plane?	◯ Yes<br>◯ No
Which bathroom did you use?	◯ Front of plane<br>◯ Rear of plane<br>◯ Both
At which door did you enter the plane?	◯ Front door<br>◯ Rear door
From which door did you exit the plane?	◯ Front door<br>◯ Rear door
Is there any further information you would like to provide?	_____________________________________
