# Supplementary material for: Transmission of SARS-CoV-2 Delta variant from an infected aircrew member on a short-haul domestic flight, Australia 2021
Source: J Travel Med. 2022 Nov 30;29(8):taac144. doi: 10.1093/jtm/taac144 (PMC9793396; doi:10.1093/jtm/taac144)
Supplement: Supplementary_Material_S3_SARS-CoV-2_taac144 [file supplementary_material_s3_sars-cov-2_taac144.docx]

**A)**


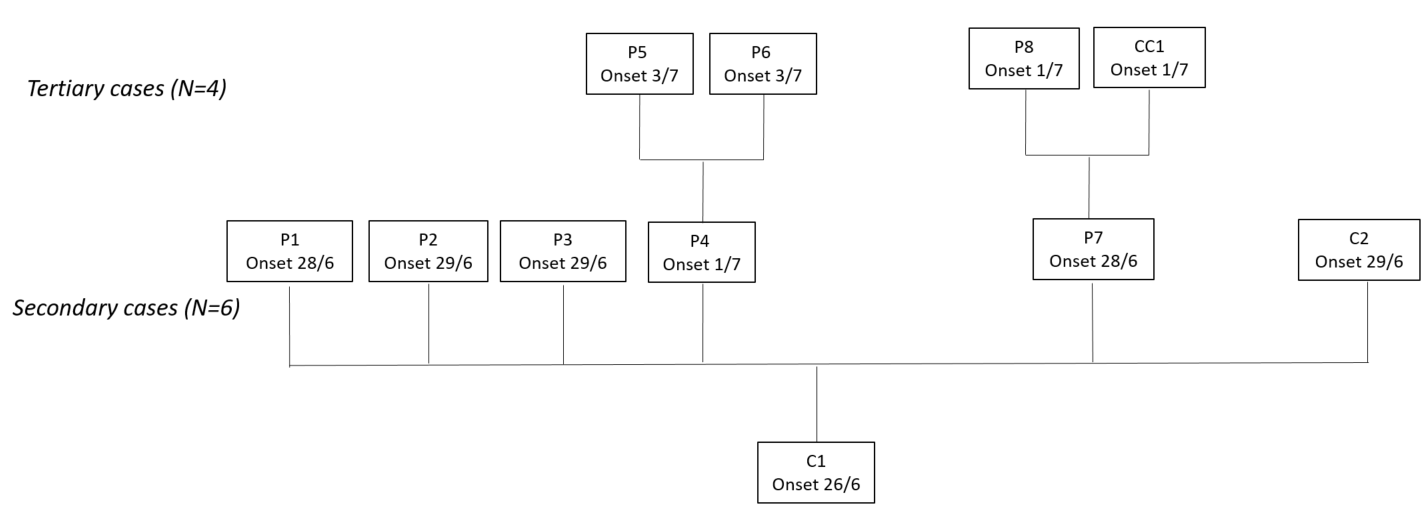


**B)**


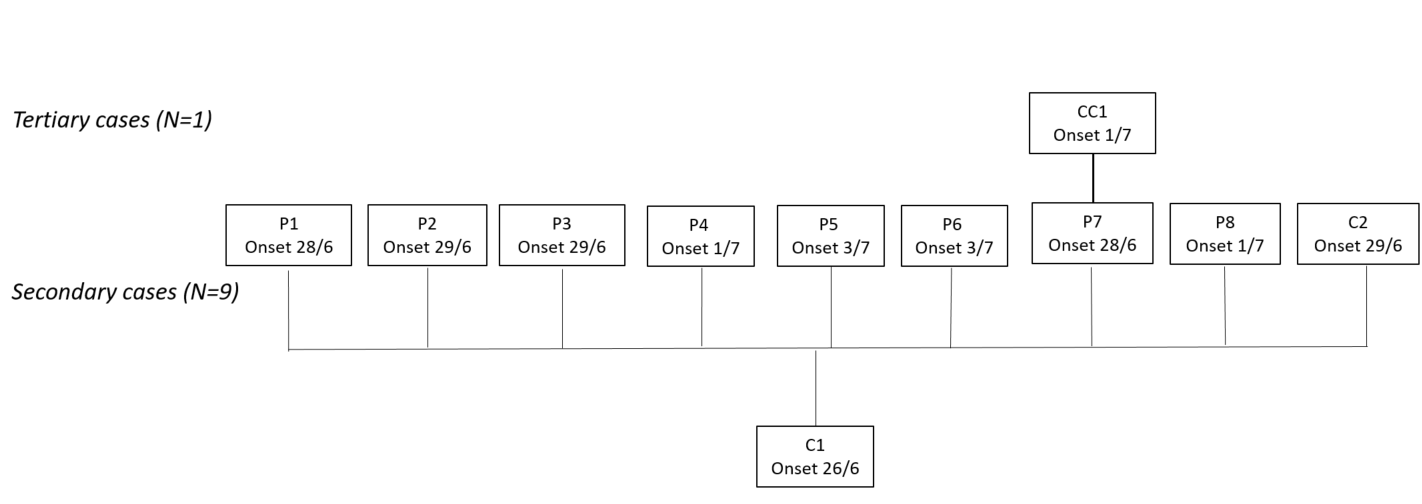


**Supplementary Material S3, Figure:**

**A)** Scenario One, where an infected crew member transmitted SARS-CoV-2 to five passenger cases and one air crew member case, with subsequent spread from infected passengers to four close contacts.

**B):** Scenario Two, where an infected air crew member transmitted SARS-CoV-2 to eight passengers and one crew member, with subsequent spread to one close contact.
