# Supplementary material for: Transmission of SARS-CoV-2 Delta variant from an infected aircrew member on a short-haul domestic flight, Australia 2021
Source: J Travel Med. 2022 Nov 30;29(8):taac144. doi: 10.1093/jtm/taac144 (PMC9793396; doi:10.1093/jtm/taac144)
Supplement: Supplementary_Material_S4_SARS-CoV-2_taac144 [file supplementary_material_s4_sars-cov-2_taac144.docx]

**Table A2:** Potential risk factors (relative risk: RR) between case and non-case passengers of Gold Coast to Sydney flight on 26 June 2021

|  |  | **Cohort Analysis** | | | |  | **Sensitivity Analysis 1** | | | |  | **Sensitivity Analysis 2** | | | |
| --- | --- | --- | --- | --- | --- | --- | --- | --- | --- | --- | --- | --- | --- | --- | --- |
|  |  | ***All 8 case passengers included*** | | | |  | ***3 potential tertiary passenger cases excluded from analysis*** | | | |  | ***3 potential tertiary passenger cases re-assigned to non-case group*** | | | |
|  |  | **No. Cases** | **No. Non-Cases** | **RR** | **p value** |  | **No. Cases** | **No. Non-Cases** | **RR** | **p value** |  | **No. Cases** | **No. Non-Cases** | **RR** | **p value** |
| **Total Passengers** | **139** | 8 | 131 |  |  |  | 5 | 131 |  |  |  | 5 | 134 |  |  |
| **Age (years)** | **Median** | 43.9 | 33.7 |  |  |  | 46.8 | 33.0 |  |  |  | 46.8 | 33.3 |  |  |
|  | **Range** | 16 - 78.9 | 0.8 - 76.7 |  |  |  | 34.3 - 78.9 | 0.8 - 76.7 |  |  |  | 34.3 - 78.9 | 0.8 - 76.7 |  |  |
| **Vaccination** | 0 dose | 8 | 105 |  | 0.767 |  | 5 | 105 |  | 0.340 |  | 5 | 108 |  | 0.349 |
|  | 1 dose | 0 | 17 |  |  |  | 0 | 17 |  |  |  | 0 | 17 |  |  |
|  | 2 dose | 0 | 9 |  |  |  | 0 | 9 |  |  |  | 0 | 9 |  |  |
|  | 0 dose | 8 | 105 |  | 0.162 |  | 5 | 105 |  | 0.268 |  | 5 | 108 |  | 0.275 |
|  | At least 1 dose | 0 | 26 |  |  |  | 0 | 26 |  |  |  | 0 | 26 |  |  |
| **Completed the survey** | | 8 | 105 |  |  |  | 5 | 105 |  |  |  | 5 | 108 |  |  |
| *** 26 missing (did not respond)** | |  |  |  |  |  |  |  |  |  |  |  |  |  |  |
| Mask | **N95/P2/Surgical** | 5 | 58 | 1.32 | 0.690 |  | 4 | 58 | 3.10 | 0.275 |  | 4 | 59 | 3.17 | 0.264 |
|  | Other e.g. cloth, cloth with filter, unknown | 3 | 47 |  |  |  | 1 | 47 |  |  |  | 1 | 49 |  |  |
|  | **Removed at some stage during flight** | 8 | 65 |  | **0.030** |  | 5 | 65 |  | 0.084 |  | 5 | 68 |  | 0.090 |
|  | Did not remove during flight | 0 | 40 |  |  |  | 0 | 40 |  |  |  | 0 | 40 |  |  |
|  | **Removed to eat (YES)** | 5 | 22 | 5.31 | **0.008** |  | 4 | 22 | 12.92 | **0.002** |  | 4 | 23 | 12.74 | **0.003** |
|  | Removed to eat (NO) | 3 | 83 |  |  |  | 1 | 83 |  |  |  | 1 | 85 |  |  |
|  | **Removed to drink (YES)** | 7 | 38 | 10.58 | **0.004** |  | 5 | 38 |  | **0.004** |  | 5 | 40 |  | **0.005** |
|  | Removed to drink (NO) | 1 | 67 |  |  |  | 0 | 67 |  |  |  | 0 | 68 |  |  |
|  | **Removed to eat or drink (YES)** | 8 | 44 |  | **0.002** |  | 5 | 44 |  | **0.011** |  | 5 | 47 |  | **0.013** |
|  | Removed to eat or drink (NO) | 0 | 61 |  |  |  | 0 | 61 |  |  |  | 0 | 61 |  |  |
| Seating | **Window seat (YES)** | 4 | 38 | 1.67 | 0.449 |  | 3 | 38 | 2.49 | 0.290 |  | 3 | 39 | 2.50 | 0.288 |
|  | Window seat (NO) | 4 | 66 |  |  |  | 2 | 66 |  |  |  | 2 | 68 |  |  |
|  |  |  | *1 missing |  |  |  |  | *1 missing |  |  |  |  |  |  |  |
|  | **Aisle seat (YES)** | 2 | 34 | 0.70 | 0.654 |  | 1 | 34 | 0.53 | 0.553 |  | 1 | 35 | 0.53 | 0.552 |
|  | Aisle seat (NO) | 6 | 70 |  |  |  | 4 | 70 |  |  |  | 4 | 72 |  |  |
|  |  |  | *1 missing |  |  |  |  | *1 missing |  |  |  |  |  |  |  |
|  | **Middle seat (YES)** | 2 | 32 | 0.76 | 0.732 |  | 1 | 34 | 0.53 | 0.553 |  | 1 | 33 | 0.57 | 0.606 |
|  | Middle seat (NO) | 6 | 72 |  |  |  | 4 | 70 |  |  |  | 4 | 74 |  |  |
|  |  |  | *1 missing |  |  |  |  | *1 missing |  |  |  |  |  |  |  |
|  | **Business Class seat (YES)** | 3 | 4 | 9.09 | **<0.0001** |  | 3 | 4 | 22.07 | **<0.0001** |  | 3 | 4 | 22.70 | **<0.0001** |
|  | Business Class seat (NO) | 5 | 101 |  |  |  | 2 | 101 |  |  |  | 2 | 104 |  |  |
|  | **Back of plane (Rows 14-30)** | 5 | 59 | 1.28 | 0.729 |  | 2 | 59 | 0.54 | 0.477 |  | 2 | 62 | 0.51 | 0.443 |
|  | Front of plane (Rows 1-13) | 3 | 46 |  |  |  | 3 | 46 |  |  |  | 3 | 46 |  |  |
| Entry Door | **Front** | 5 | 60 |  | 0.057 |  | 4 | 60 |  | 0.088 |  | 4 | 61 |  | 0.090 |
|  | Back | 0 | 45 |  |  |  | 0 | 45 |  |  |  | 0 | 45 |  |  |
|  |  | *3 missing |  |  |  |  | *1 missing |  |  |  |  | *1 missing | *2 missing |  |  |
| Exit Door | **Front** | 3 | 61 | 1.08 | 0.933 |  | 3 | 61 | 2.11 | 0.500 |  | 3 | 61 | 2.16 | 0.487 |
|  | Back | 2 | 44 |  |  |  | 1 | 44 |  |  |  | 1 | 45 |  |  |
|  |  | *3 missing |  |  |  |  | *1 missing |  |  |  |  | *1 missing | *2 missing |  |  |
| Bathroom | **Used** | 1 | 9 | 2.50 | 0.385 |  | 1 | 9 | 3.30 | 0.264 |  | 1 | 9 | 3.33 | 0.260 |
|  | Did not use | 4 | 96 |  |  |  | 3 | 96 |  |  |  | 3 | 97 |  |  |
|  |  | *3 missing |  |  |  |  |  |  |  |  |  |  |  |  |  |
| Hand Sanitizer | **Used** | 0 | 46 |  | 0.130 |  | 0 | 46 |  | 0.379 |  | 0 | 46 |  | 0.387 |
|  | Did not use | 3 | 59 |  |  |  | 1 | 59 |  |  |  | 1 | 61 |  |  |
|  |  | *5 missing |  |  |  |  |  |  |  |  |  |  |  |  |  |
